# Fast characterisation of cell-derived extracellular vesicles by nanoparticles tracking analysis, cryo-electron microscopy, and Raman tweezers microspectroscopy

**DOI:** 10.3402/jev.v1i0.19179

**Published:** 2012-11-21

**Authors:** Irène Tatischeff, Eric Larquet, Juan M. Falcón-Pérez, Pierre-Yves Turpin, Sergei G. Kruglik

**Affiliations:** 1 Laboratoire Jean Perrin, FRE 3231 CNRS, Université Pierre et Marie Curie, Paris, France; 2 Laboratoire d'Enzymologie et Biochimie Structurales, UPR 3082 CNRS, Gif-sur-Yvette, France; 3 CIC bioGUNE, CIBERehd, Metabolomics Unit. IKERBASQUE, Basque Foundation for Science, Bilbao, Spain

**Keywords:** extracellular vesicles, nanoparticle tracking analysis, cryo-electron microscopy, Raman tweezers microspectroscopy, urinary exosomes, Dictyostelium discoideum

## Abstract

The joint use of 3 complementary techniques, namely, nanoparticle tracking analysis (NTA), cryo-electron microscopy (Cryo-EM) and Raman tweezers microspectroscopy (RTM), is proposed for a rapid characterisation of extracellular vesicles (EVs) of various origins. NTA is valuable for studying the size distribution and concentration, Cryo-EM is outstanding for the morphological characterisation, including observation of vesicle heterogeneity, while RTM provides the global chemical composition without using any exogenous label. The capabilities of this approach are evaluated on the example of cell-derived vesicles of *Dictyostelium discoideum*, a convenient general model for eukaryotic EVs. At least 2 separate species differing in chemical composition (relative amounts of DNA, lipids and proteins, presence of carotenoids) were found for each of the 2 physiological states of this non-pathogenic microorganism, that is, cell growth and starvation-induced aggregation. These findings demonstrate the specific potency of RTM. In addition, the first Raman spectra of human urinary exosomes are reported, presumably constituting the primary step towards Raman characterisation of EVs for the purpose of human diseases diagnoses.

The field of cell-derived extracellular vesicles (EVs) is expanding very rapidly, both in biology and in medicine (1,2). In the absence of a consensus on the nomenclature for various kinds of EVs, the term “EVs” is used throughout this article to designate secreted vesicles in general, with no qualification based on defined criteria. For well known subtypes, like exosomes, the more precise qualification is mentioned, when appropriate.

The need for a better characterisation of EVs in the 50–500 nm size range is, presently, an important challenge. Biological nanovesicles are mostly out of scope of usual flow cytometry (3–5), devoted to the statistical analysis of components larger than 1 µm in diameter. However, recent improvements allow flow cytometry analysis of nano-sized EVs (5,6) and this fast technique keeps its statistical advantage for EVs characterisation, especially when used with fluorescent labels (3). Nanoparticle tracking analysis (NTA) is a rapidly emerging technique (7) for the fast characterisation of the size distribution and concentration of EV populations. NTA has been favourably evaluated, when compared to the other techniques used for that purpose (3,7,8).

Cryo-electron microscopy (Cryo-EM), which appeared 3 decades ago (9), is a powerful tool for imaging biological EVs in their native aqueous environment, without any added fixatives. Surprisingly, it has seldom been used for that purpose up to now (10,11), although Cryo-EM studies of viruses (12–14), or cells (15,16), are widespread.

Raman tweezers microspectroscopy (RTM), with laser-mediated tweezing of the object under study using a microscope objective with a high numerical aperture, provides genuine Raman fingerprints of the sample chemical constituents in a time range from seconds to minutes, without any exogenous marker or sample treatment. This approach has already been applied to bacteria (17), spores (18), tumour cells in suspension (19) and to liposomes of a sub-micrometer size (20,21). The first biological applications of RTM for analysis of nerve cell organelles emerged in 2002 (22). However, although being a quite promising technique for virology (23) and for the study of all virus size-related EVs, RTM is just entering the field of cell-derived EVs. The first Raman spectrum of EVs was reported in 2009 (10), and the utilisation of Raman microspectroscopy for vesicle characterisation was theoretically discussed in 2010 (3).

We applied jointly these 3 techniques, that is, NTA, Cryo-EM and RTM, for a characterisation of cell-derived vesicles of *Dictyostelium discoideum*, a convenient general model for eukaryotic EVs, by studying the concentration and size distribution, the morphology and the global chemical composition, respectively. *D. discoideum* is a unicellular eukaryotic microorganism (see http://dictybase.org/), which was chosen in 1999 by the NIH (United States) as a pertinent model for biomedical research. *D. discoideum* EVs act as detoxifying agents, but they also present the signatures of a constitutive vesicle secretion process of *D. discoideum* cells (24), occurring during both cell growth and starvation-induced early development (aggregation). Considering that *Dictyostelium* cells are much easier to manipulate than human cells, they can be used as a useful model to unravel some of the many biological functions of eukaryotic EVs. This was proposed at the 1st Annual Meeting of the International Society for Extracellular Vesicles (ISEV) 2012 (25). Advantage is taken of the two well-separated physiological states of this microorganism, corresponding to cell growth and starvation-induced aggregation, to test the ability of RTM to specifically characterise each *D. discoideum* EVs subpopulation.

Furthermore, as a proof of principle, preliminary RTM data are presented on urinary exosomes from 4 healthy people, suggesting the use in the future of the Raman Tweezers technique as a potent, rapid and label-free disease diagnostic tool in physiological fluids.

## Materials and methods

### D. discoideum cell cultures and preparation of extracellular vesicles

*Dictyostelium* cells, Ax-2 strain, are grown in suspension in the dark, on a gyratory shaker (150 rpm) at 22°C, in HL5 semi-defined medium (ref. HLBO101, Formedium, United Kingdom), added with (7.8% v:v) 0.20 µm filter-sterilised D-glucose (1 M) and 1% antibiotics [(penicillin (50 U/ml) and streptomycin (50 µg/ml) (Biomedia, Boussens, France)]. HL5 semi-defined medium is free of vesicles (24). For proper oxygenation, each suspension is grown in an Erlenmeyer containing 5 times the suspension volume.

For growth, cells are inoculated at a density of 5 to 10×10^5^ cells ml^−1^ and, either in the exponential phase after 24 hours of growth, or in the early stationary phase after 48 hours of growth, EVs are concentrated from the growth medium. This is performed by 3 successive differential centrifugations (700×g for 5 minutes at 20°C; 2,000×g for 10 minutes at 20°C and 12,000×g for 30 minutes at 4°C), as described in (10). EVs, present in the 12,000×g pellet, are resuspended in a phosphate-buffered solution (PBS) [pH 7.4 without calcium and magnesium (Gibco)], with a 20 to 100 volume concentration factor (X), relative to the volume of the 2,000×g supernatant. The 700×g and 2,000×g centrifugations are performed in single-use 15 ml tubes on an Eppendorf 5804R centrifuge. The 12,000×g centrifugation for 30 minutes is generally performed in 1.5 (or 2 ml) Eppendorf tubes in a Mikro 22 R centrifuge and the pellets are pooled for the final resuspension in PBS. For comparison, a 12,000×g centrifugation has also been performed during 1 hour at 4°C in 30 ml Corex tubes in a Sorval RC5C centrifuge equipped with a SA-600 rotor.

For starvation, cells in the early stationary phase of growth in suspension are centrifuged at 700×g for 5 minutes at 20°C, washed twice in KK_2_ 17 mM phosphate buffer (pH 6.8) with the same centrifugation speed and resuspended in the same buffer at 4×10^7^ cells ml^−1^.The cell suspension is kept 22 hours in an Erlenmeyer, containing 5 times the suspension volume for proper oxygenation, on a gyratory shaker (150 rpm) at 22°C. Then, EVs are concentrated from the starvation medium by differential centrifugations, as described above.

The *Dictyostelium* EVs, prepared from either growth or starvation medium, are stable for months in PBS, when kept at 4°C until use (10,26).

### Preparation and characterisation of urine exosomes

Urinary exosomes were isolated from 50 ml of urine samples from human healthy individuals after 8-hours fasting period, as described in (27). Briefly, collected urine samples are centrifuged for 30 minutes at 1,500×*g*. The resultant supernatants are subjected to filtration on 0.22 µm pore filters, followed by ultracentrifugation at 10,000×*g* and 100,000×*g* for 30 minutes and 60 minutes, respectively. The resulting pellets are suspended in PBS, pooled and again ultracentrifuged at 100,000×*g* for 60 minutes. The final pellet of urinary exosomes is suspended in 150 µl of PBS and stored at −80°C.

### NanoSight NTA

The measurements on *D. discoideum* EVs (Table I) were performed with a NanoSight LM10 equipment (NanoSight, Amesbury, United Kingdom). This set-up is based on a conventional optical microscope, which uses a (40 mW) 640 nm laser light source to illuminate nano-scale particles within the size range of 10–100 nm. The 0.3 ml sample is introduced to the viewing unit with a disposable syringe. Enhanced by a near perfect black background, particles appear individually as point-scatterers moving under Brownian motion. Polydisperse and multimodal systems are instantly recognisable and quantifiable. The image analysis NTA software allows the automatic tracking and sizing of nanoparticles on an individual basis. It offers real-time dynamic nano-particle visualisation, particle-by-particle analysis, particle counting and sizing, and particle size distributions. The concentration and size distribution of the urinary vesicles (mostly exosomes), smaller than 240 nm in size, were analysed by NTA with NanoSight equipment, previous to RTM measurements (Table II).

**Table I:** NTA measurements of *D. discoideum* extracellular vesicles

Sample	a^1^	b^1^	c^1^
NanoSight Dilution	1,000	5,000	100
Mean diameter (nm)	253	259	163
Mode (nm)	150	195	158
Diameter SD (nm)	176	140	58
Concentration (particles/ml)	3.77×10^8^	3.97×10^8^	9.96×10^8^
Completed tracks	748	631	4,377
EVs/ml in the medium	1.89×10^10^	1.99×10^10^	9.96×10^10^

1 Samples a and b, prepared from the growth medium after 48 hours growth of *D. discoideum* cells (see Materials and methods), were concentrated in PBS by factors of 20 and 100, respectively. The 12,000×g supernatant (c), corresponding to EVs (b), was used as obtained after the 12,000×g centrifugation.

**Table II:** NTA characterisation of exosomes from human urinary samples

Sample	Mean diameter (nm)	Diameter SD (nm)	Concentration (particles/ml)
P2_1_M (male)	172	72	3.70×10^9^
P2_10_F (female)	134	54	1.29×10^11^
P2_11_M (male)	144	54	1.74×10^11^
P2_15_F (female)	131	51	2.59×10^11^

### Transmission cryo-electron microscopy

A 4 µl droplet of the vesicle suspension was applied to a 200 mesh *R 2/2* Quantifoil^®^ holey-carbon grid. Excess of solution is removed with Whatman paper and the grid is rapidly plunged into liquid ethane (28,29) and transferred under liquid nitrogen into the microscope using a side entry nitrogen-cooled Gatan 914 cryoholder. Sample analysis was carried out under a JEOL JEM-2200FS (Cs=1.4 mm) transmission cryoelectron microscope (PICT-IBiSA Imaging Facility) with an acceleration voltage of 200 kV, a nominal magnification of 54,804×estimated with tobacco mosaic virus as a reference (30) and defocus ranging from −1.2 to −2.5 µm, accurately determined using enhanced power spectra (31). Images were recorded under low dose conditions (10 electrons per A^2^ per second) with a *2k*×*2k* Gatan Utrascan™ 1000 CCD camera.

### Raman tweezers setup

Raman spectra were recorded using a home-built near-infrared (NIR) Raman tweezers setup. The main idea consists of utilising the same strong laser radiation for both optical trapping of the vesicles dispersed in aqueous medium and excitation of Raman scattering from the vesicle's constituent molecules. In our setup, laser excitation light at 780 nm is provided by continuous-wave Ti:Sapphire laser (Spectra Physics, model 3900S) pumped by argon-ion laser (Spectra Physics Stabilite 2017). The laser beam is focused from above by water-immersion infinity-corrected objective (Olympus LUMFL 60X, NA=1.1) brought into contact with a droplet (~50–100 µL) of the sample solution put onto a calcium fluoride (CaF_2_) substrate. The focal waist of intense laser radiation with an average power of ~100 mW serves as an optical trap and is located inside the sample solution at a distance of about 2 mm above the substrate surface. Such excitation geometry assures that the signal originates from the trapped vesicles, with no hindering contribution from the environment (except for the PBS signal), even without the use of a true confocal optical arrangement that substantially reduces the intensity of the Raman signal.

The same objective is used to collect the Raman signal in a backscattering geometry and to deliver it onto the spectrograph (Acton SpectraPro 2500i) coupled with a deep-depletion back-illuminated NIR CCD (Princeton Instruments SPEC-10 400BR/LN). Raman light is focused onto the spectrograph's entrance slit (semi-confocal configuration, slit width 30–40 μm) by an achromatic lens with *f*=75 mm. The Raman signal is separated from laser stray light by 2 Semrock RazorEdge long-pass filters (grade “U”). One filter, with λ_laser_=780 nm, is placed normally to the optical beam just before the focusing lens; another one, with λ_laser_=830 nm, is used as a dichroic beam splitter at an angle of incidence 45°: it reflects laser light at 780 nm and transmits all the wavelengths longer 785 nm. Spectral resolution in all Raman experiments was about 5 cm^−1^. A frequency calibration was performed using Raman lines of toluene with ±2 cm^−1^ absolute accuracy and relative frequency position accuracy better than ±1 cm^−1^. Raman spectra were acquired using WinSpec software; further data treatment was performed using Igor Pro for Windows software.

### Raman spectra acquisition and data treatment

The informative signal from the vesicles in the sample volume arises as additional spectral features appearing on top of the PBS Raman spectrum, as soon as the vesicles are trapped at the focus of the laser beam. The process of vesicles trapping is rather stochastic and the resulting Raman signal depends on various parameters such as the vesicle molecular composition, size, concentration, density and the laser power.

Before the study on EVs, we performed a preliminary investigation of the particularities of the RTM signal using a suspension of model DOPC liposomes of various known sizes, solutions of bovine serum albumin (BSA) and calf-thymus DNA, as a function of sample concentrations. Briefly, our setup is capable of trapping and detecting a very weak but distinguishable Raman signal from a single empty liposome of 200-nm size, while several (~5–10) trapped vesicles in the 50–200 nm size range are required for a reliable liposome characterisation. No Raman signal was detected from small BSA and DNA aggregates probably because they are not trapped in our RTM configuration. The detailed description of this characterisation study will be published elsewhere; here we report the first RTM results on EVs relevant from the general scientific/medical viewpoints.

Raman spectra are acquired every 3 seconds (for *D. discoideum* EVs) or every 6 seconds (for urine exosomes) during a sequence of 20 consecutive measurements: this constitutes one “experimental set”. Measurements keep going until the event of optical trapping of at least several vesicles in the focus of the microscope objective (so-called “vesicle set”); after this event, a few more experimental sets are sequentially acquired for a better signal averaging. The transition from one vesicle set to another is accomplished by blocking the laser beam for 10 seconds, the trapped particles in the measurable volume being then exchanged by Brownian motion.

The process of data treatment consists of the following successive steps:

- All of the recorded raw Raman spectra are visually inspected and properly selected, to eliminate the spectra without (or with very minor) vesicles contribution, with high background and poor quality.
- The selected raw Raman spectra are averaged and normalised on the water bending band around 1,640 cm^−1^.
- The contribution from PBS is subtracted.
- The resulting spectra are smoothed by 2 (for *D. discoideum* EVs) or 3 (for urinary exosomes) adjacent pixels, to minimise the etaloning effect in back-illuminated NIR-CCD detector.
- Finally, in some cases, a slowly changing background originating from strong stray Rayleigh scattering is subtracted in using the cubic spline interpolation from Igor Pro software.

Note that no further mathematical treatment, by any multivariate algorithm of the resulting Raman spectra, was performed in the present study.

## Results and discussion

### Size distribution and concentration of D. discoideum extracellular vesicles

**Fig. 1:**
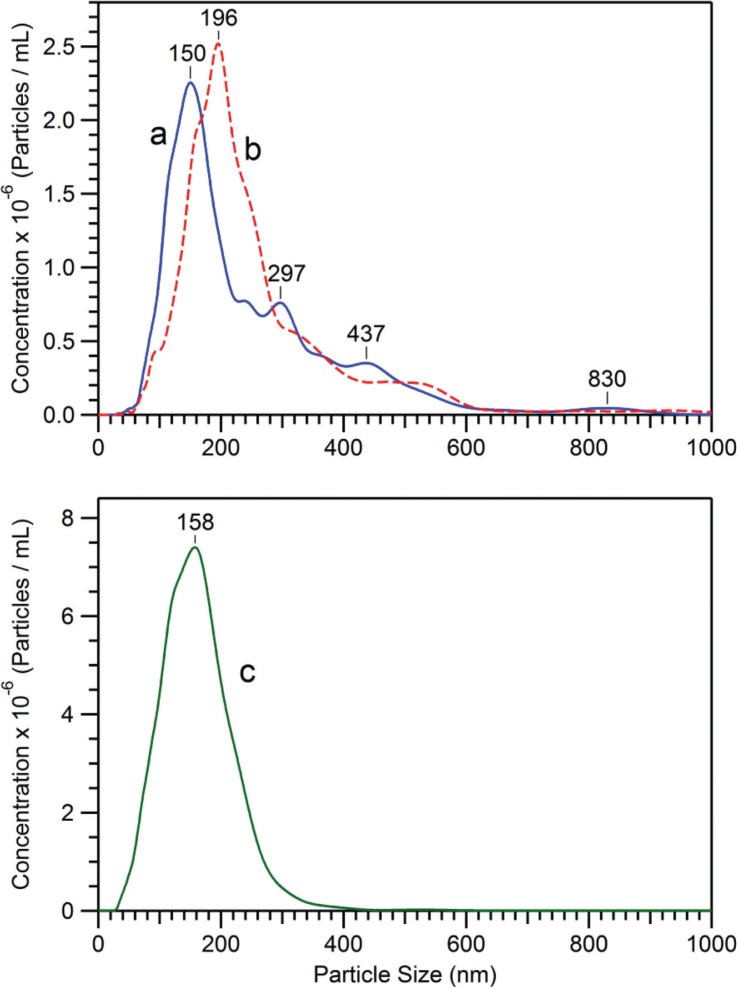
Size distribution and concentration of *D. discoideum* EVs samples (Table I), as measured by NTA. EVs were prepared from growth medium after 48 hours of cell growth, either with the usual conditions for the 12,000×g centrifugation (in Eppendorf tubes) (a), or with the 12,000×g centrifugation performed in 30 ml tubes (see Materials and methods) (b). The curve (c) corresponds to EVs remaining in the 12,000×g supernatant, which relates to sample (b) EVs. The 12,000×g pellets (a and b) are concentrated in PBS by factors of 20 and 100, respectively, whereas the 12,000×g supernatant (c) is as obtained after centrifugation.

Figures 1a and 1b show the size distributions and concentrations of 2 different preparations of *D. discoideum* EVs obtained after 48 hours of cell growth (see also Table I), as measured by NanoSight NTA. The vesicles were either prepared using the usual conditions for the 12,000×g centrifugation (in Eppendorf tubes) (a), or with the 12,000×g centrifugation performed in 30 ml tubes (b) (see Materials and methods). Although the concentrations of EVs in the growth medium are almost the same for the 2 preparations, the size distributions appear to be different. It means that different conditions for the 12,000×g centrifugation can introduce a biased evaluation of the genuine size distribution of the vesicles in the extracellular medium.

Besides this, it is also noteworthy that the 12,000×g pelleted vesicles represent less than 17% of the total amount of vesicles present in the extracellular medium of *D. discoideum* cells after 48 hours of growth. This is illustrated in Fig. 1c obtained from the 12,000×g supernatant of the EVs sample, showing that many smaller EVs remain in this supernatant, and have a less dispersed size distribution (see Table I). This also means that the complete spectrum of all of the cell-derived vesicles has to be sought directly in the cell-free extracellular medium (i.e. obtained at 2,000×g), this total population being mandatory for understanding all of the physiological functions of EVs, without any artificial restriction due to their mode of preparation.

This should be kept in mind when discussing the general EVs preparations and nomenclatures. For a given cell model, the actual EVs field is probably unexpectedly large. It is quite important to precisely define the protocols of preparation for the different EVs subpopulations to be analysed, taking care not to exclude any EVs subpopulation.

### Morphology and heterogeneity of D. discoideum extracellular vesicles

**Fig. 2:**
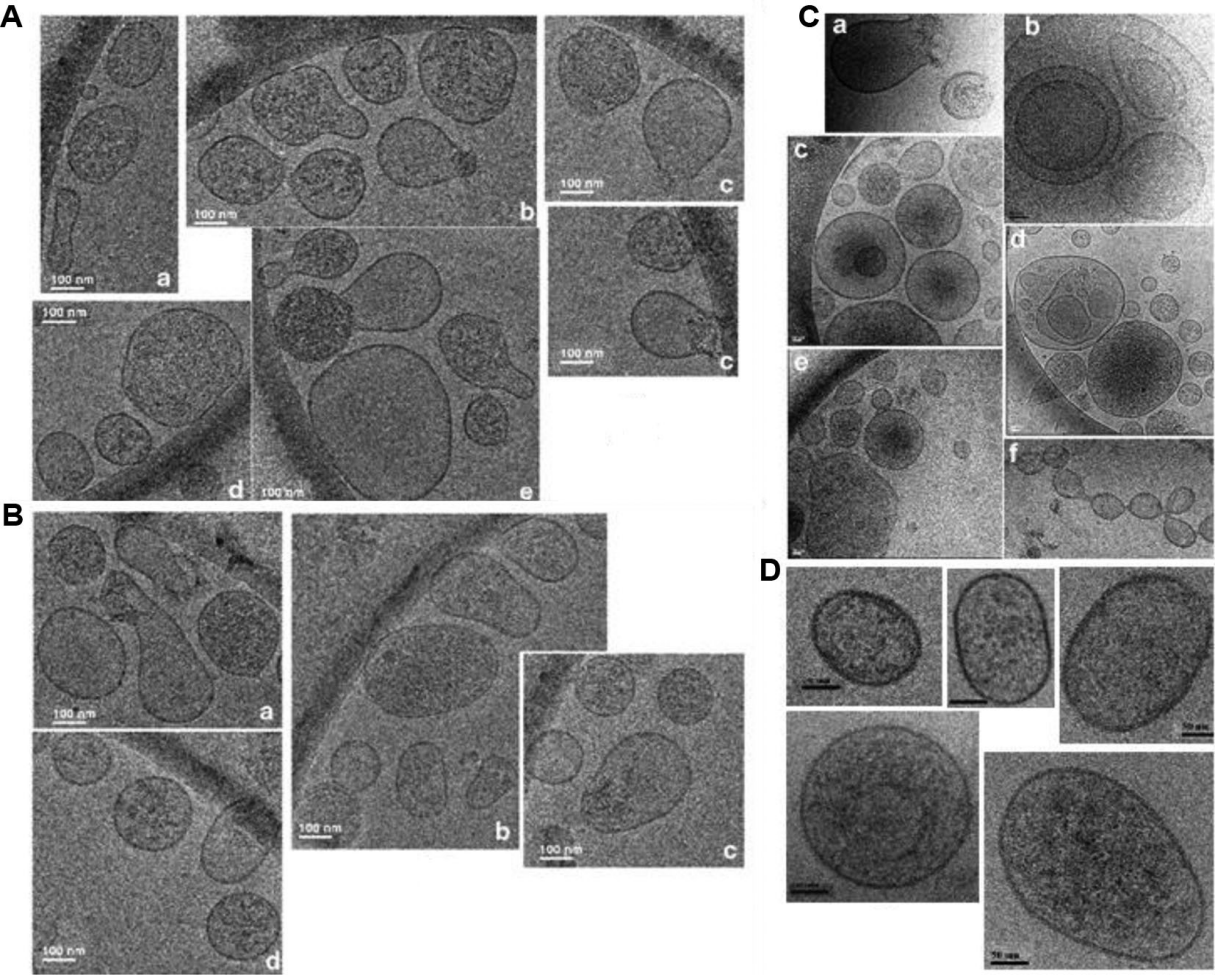
Observation of *D. discoideum* EVs by Cryo-EM. (A) EVs obtained after 24 hours of cell growth; (B) EVs obtained after 48 hours of cell growth. (C) Some rare EVs configurations, conserved in the vitreous ice environment, observed during growth, showing broken vesicles (a), big EVs inserted inside larger vesicles (b, d) or small EVs into multivesicular bodies-like vesicles (c, e) and EVs prone to fuse (c, e, f). Figure 2D shows 5 different images of EVs obtained from the starvation medium of *D. discoideum* cells (see Materials and methods). Bars: 100 nm for A and B, 50 nm (a–e) or 100 nm (f) for C and 50 nm for D.

Figure 2A obtained by Cryo-EM, as collected from 5 different images of the same cryo-sample, shows the morphology and heterogeneity of *D. discoideum* EVs, obtained after 24 hours of cell growth. Figure 2B, as collected from 4 different images, depicts the same heterogeneity of EVs, obtained after 48 hours of cell growth. A previous observation by Cryo-EM on 59 *D. discoideum* EVs showed that about 80% of them had an average diameter within a 50–150 nm range (10), that is, in the same range as the statistically more relevant NTA measurements. Qualitatively, NTA and Cryo-EM are complementary, NTA being a more convenient technique for a straightforward observation of EVs size distribution and concentration, while Cryo-EM is most valuable for a precise observation of EVs morphology and heterogeneity (Figs. 2A, B), and of some rare EVs configurations, conserved in a vitreous ice environment, as depicted in Fig. 2C. It should be noted that EVs generally are not empty, as shown on broken vesicles (Fig. 2C a ); they can be either big and inserted inside larger vesicles (Fig. 2C b, d) or small and inserted into multivesicular bodies-like vesicles (Fig. 2C c, e). In Cryo-EM, the thickness of the vitreous ice film is estimated between 60 and 120 nm. A contrast analysis of the images reveals that the vesicles of interest are quite close to the same focalisation plane, that is, inserted into and not below or above each other. This would, however, deserve further study by molecular tomography for confirmation. EVs can also be prone to fuse (Fig. 2C c, e, f). Figure 2D shows a composition from 5 different images of EVs obtained from the starvation medium of *D. discoideum* cells: they seem to be more regular in shape and contour, than those observed during growth (see Figs. 2A, B, C). However, this observation should be confirmed by additional Cryo-EM measurements, to be statistically significant.

### Raman signatures of D. discoideum extracellular vesicles

**Fig. 3:**
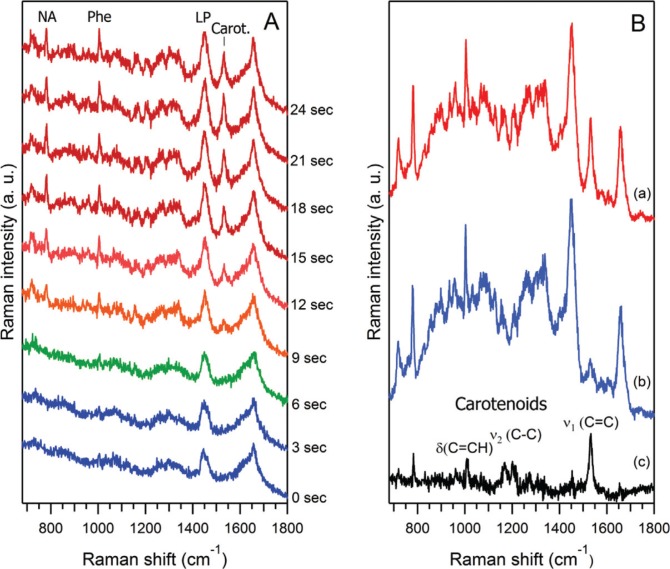
Raman spectra of optically trapped vesicles released from *D. discoideum* cells in the starvation phase. (A) The time sequence of raw Raman spectra recorded every 3 seconds, for one particular vesicle set. The event of vesicle(s) trapping is seen within the time window from 6 to 9 seconds, by the appearance of characteristic Raman bands of nucleic acids (NA, ~783 cm^−1^), phenylalanine (Phe, ~1,005 cm^−1^) and the intensity increase of the lipids/proteins marker of CH_2_ groups deformations (LP, ~1,450 cm^−1^). Note also the appearance and gradual increase of the prominent carotenoids Raman marker at ~1,533 cm^−1^. (B) Raman spectra for 2 different vesicle sets (a, b) and their difference spectrum (c = a − b×0.88) corresponding to the characteristic Raman spectrum of carotenoids whose major bands [ν_1_, ν_2_, δ(C = CH)] are indicated at trace (c). Raman spectra (a) and (b) have been obtained from raw spectra after subtraction of the PBS contribution. The factor 0.88 was found empirically to emphasise the carotenoids’ contribution in spectrum (c).

Up to now, the chemical characterisation of *Dictyostelium* EVs, by techniques such as thin liquid chromatography for lipid analysis (24) or proteomics for proteins analysis (10), has been performed on bulk preparations and were, therefore, unable to take into account the individual heterogeneity of the vesicles. In contrast, RTM is a technique of choice for a global chemical/structural study, in a time scale of a few seconds, of individually trapped EVs, and for monitoring variations of some vesicular components as a function of the acquisition time. This is illustrated with EVs prepared from the medium of *D. discoideum* cells after a 22-hour starvation period (Fig. 3). In the raw data for one particular vesicle set (Fig. 3A), the early (0s to 6s) Raman spectra contain contribution solely from the medium where vesicles are floating. The event of EVs trapping is seen within the time interval from 6 to 9 seconds, by the appearance of characteristic Raman bands of nucleic acids (NA,~783 cm^−1^), phenylalanine (Phe,~1,005 cm^−1^) and the intensity increase of the lipids/proteins marker of CH_2_ groups deformation (LP, ~1,450 cm^−1^). The characteristic Raman features of the trapped vesicle (or a small vesicle-cluster) persist as long as the vesicle remained optically trapped. It should be noted that, from the intensity of Raman signals and taking into account the inhomogeneity of EVs size distribution, it is difficult to estimate whether one big vesicle or a cluster of several smaller vesicles have been optically trapped. Our preliminary RTM study on empty liposomes of 50–200 nm size suggests that a rather strong, readily distinguishable Raman signal (as in Fig. 3) originates from a cluster of at least several (2–10) vesicles.

By manually moving the ~100 µl sample droplet or by temporarily blocking the laser excitation, to focus on another EV (or vesicle-cluster), it appears that the vesicles are indeed heterogeneous, with various proportions of the chemical compounds relative to each other, see for instance Fig. 3B. As a preliminary study, about 10 EVs were observed, with 20 sets of 20×3s acquisitions for each EV.

One more potentiality of RTM is to identify any minor, newly appearing component during the raw measurements, by subtracting the previous nearest set from the set showing the new component, as shown in Fig. 3B. The Raman spectra for 2 different vesicles sets (a, b), are obtained from raw spectra after subtraction of the PBS contribution. Their difference spectrum (c=a−b×0.88) corresponds to the characteristic Raman spectrum of carotenoids (32), whose major bands (ν_1_, ν_2_, δ(C=CH)) are indicated at trace (c). The appearance and gradual increase of the prominent carotenoids Raman marker at ~1,533 cm^−1^ is observed in Fig. 3A. The identification of carotenoids in starvation EVs fits well with an ancient observation of carotenoids appearing into *D. discoideum* cells during starvation and responsible for the observed yellow colour of the fruiting bodies (33).

**Fig. 4:**
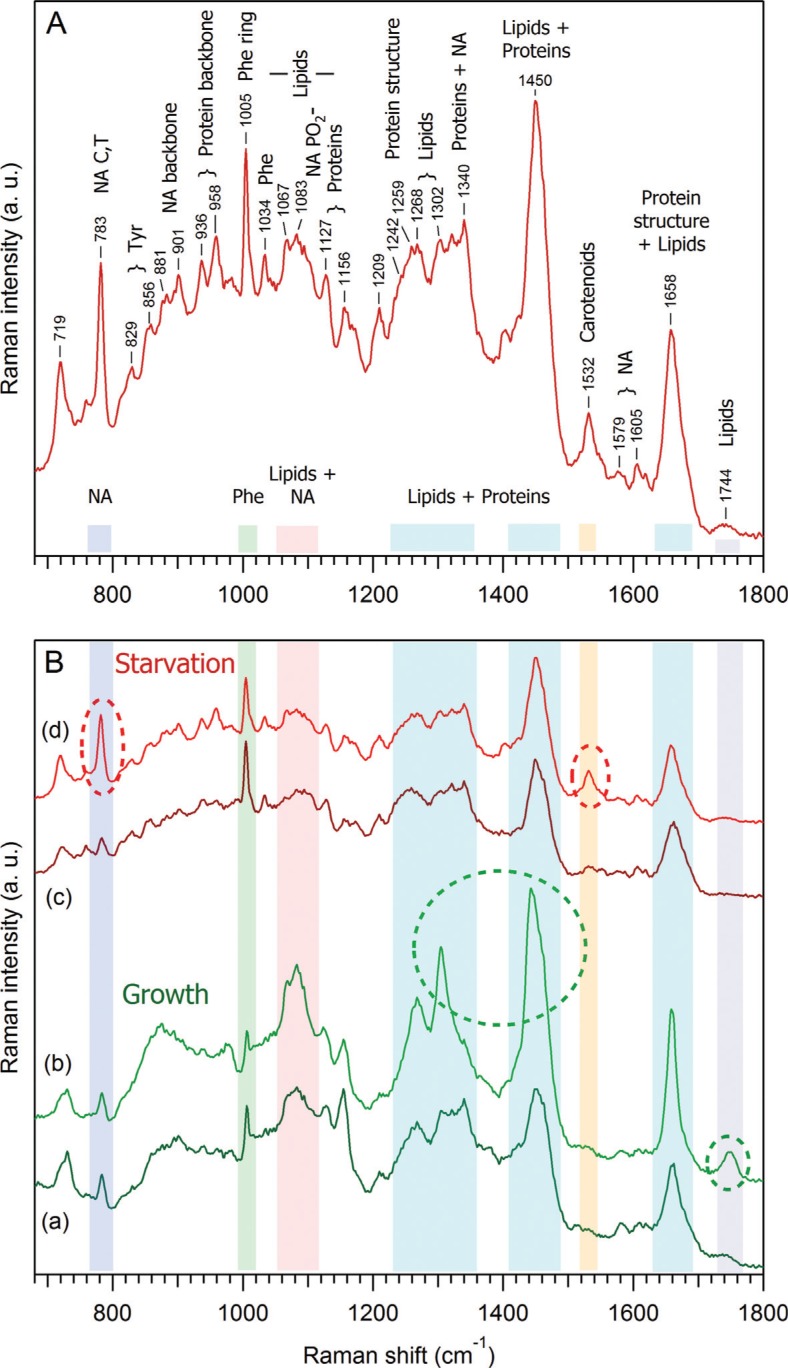
Comparison of the Raman spectra of *D. discoideum* growth and starvation EVs. (A) Proposed interpretation of Raman spectrum originated from vesicles released from *D. discoideum* in the starvation phase. This spectrum was obtained by averaging 15 different sets of vesicles, with a total accumulation time of 14 minutes, then subtraction of the PBS contribution, and smoothing by 2 adjacent pixels. (B) Variability of Raman spectra of *D. discoideum* vesicles during growth (a, b) as compared to starvation phase (c, d). Within the same phase, one can qualitatively distinguish at least 2 different species: during growth, species (b) is characterised by an increased amount of lipids as compared to species (a), and during starvation, species (d) contains an increased amount of nucleic acids and carotenoids as compared to species (c). The prominent spectral differences are highlighted by dashed oval curves.

Figure 4A shows the proposed interpretation of Raman spectrum originated from the EVs released from *D. discoideum* cells in the starvation phase. The spectrum was obtained by averaging 15 different sets of vesicles, with a total accumulation time of 14 minutes, then subtracting the PBS contribution and smoothing by 2 adjacent pixels. The main Raman signatures of lipids, proteins and NA are identified. Moreover, as shown in Fig. 4B (c, d), it is possible to characterize 2 major species of EVs for the starvation phase, one with prominent NA and carotenoids signatures (d) and the other with much lower NA contribution (c). The same treatment has been performed for EVs prepared from the extracellular medium of *D. discoideum* cells after 48 hours of growth (Fig. 4B a, b). At least 2 EVs species are also present during the growth phase, being different mainly by the amount of lipids: species (b) is characterised by an increased amount of lipids, as compared to species (a). As shown in Fig. 4B, it is also clear that *D. discoideum* vesicles externalised during growth (Fig. 4B a, b) are different from those externalised during early starvation-induced development (Fig. 4B c, d). The first structural identification of the main Raman components of EVs during starvation (Fig. 4A), along with the comparison (Fig. 4B) of EVs during two different physiological states of *D. discoideum* cell life, demonstrate the potential of RTM for global chemical/structural analysis of EVs.

### Raman tweezers microspectroscopy of exosomes from human urine samples

**Fig. 5:**
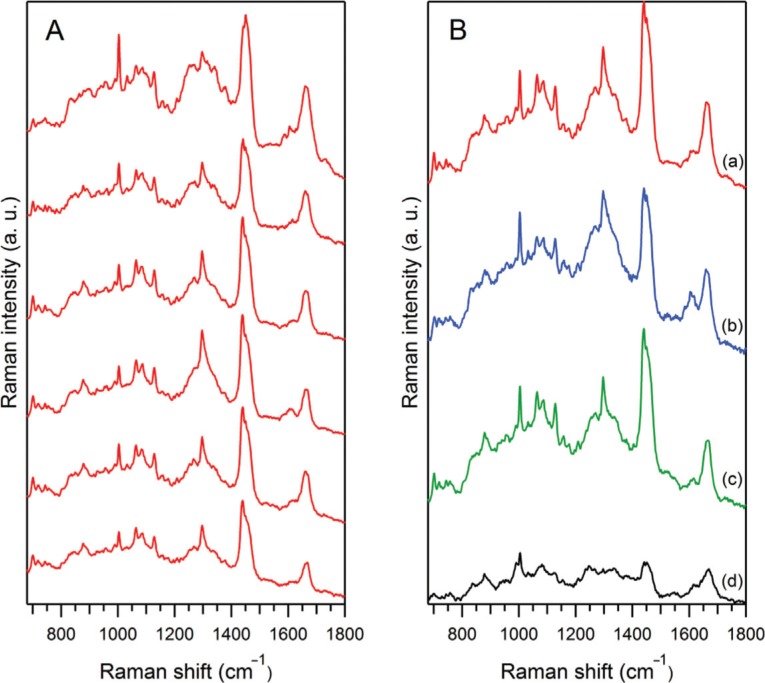
Raman spectra of optically trapped urinary exosomes from healthy human samples (see Table II). (A) Similarity/variability of spectra from 6 different vesicles sets, for the same sample “P2_11_M” (a), each averaged over ~50 raw Raman spectra. (B) Raman spectra for 4 different exosome samples: “P2_11_M” (a) (averaging of 303 raw spectra), “P2_10_F” (b) (266 raw spectra), “P2_15_F” (c) (287 raw spectra) and “P2_1_M” (d), (140 raw spectra). All spectra were corrected for the PBS contribution, the slowly changing background from Rayleigh scattering and smoothed by 3 adjacent pixels (see Materials and methods).

To test the applicability of RTM as a possible fast and label-free diagnostic tool in physiological fluids, a preliminary assay was also performed with human urine exosomes, prepared from 4 healthy people and characterised by NTA measurements (Table II). For sample, P2_11_M, Fig. 5A shows the similarity/variability of Raman spectra from 6 different vesicle sets. Each “experimental set spectrum” is obtained by averaging about 50 raw Raman spectra obtained with an accumulation time of 6 seconds each, after the event of optical trapping. Figure 5B shows the averaged results for the 4 different exosomes samples. Raman spectra were obtained by averaging of raw spectra: “P2_11_M” (a) (averaging of 303 raw spectra), “P2_10_F” (b) (266 raw spectra), “P2_15_F” (c) (287 raw spectra) and “P2_1_M” (d) (140 raw spectra). Whereas the exosome concentrations of 3 out of 4 samples, ranging between 1.3 and 2.6×10^11^ EVs/ml (see Table II), were convenient for our measurements, the exosome concentration for sample P2_1_M (d) (3.7×10^9^/ml) was at the limit of the detection capability of our setup.

This is, indeed, the first experiment showing the feasibility of RTM for obtaining direct Raman signatures of urinary EVs of less than 240 nm in size (mostly exosomes), prepared from a physiological fluid. More work is to be done, first on more numerous samples from healthy people, to understand the individual variability, second on samples from patients suffering a given disease, for an eventual diagnostic purpose.

## Conclusion

The approach of a rapid characterisation of EVs in the 50–500 nm size range is proposed by the joint use of 3 complementary methods, NTA, Cryo-EM and RTM, with easy sample preparation and without the use of any added label, which is in turn complementary to other more informative but much more time consuming techniques, such as proteomics, genomics, lipidomics or metabolomics. In particular, the capabilities of RTM were demonstrated on the example of cell-derived vesicles of *D. discoideum*, where at least two separate species differing in global chemical composition (relative amounts of DNA, lipids and proteins, presence of carotenoids) were found for both of its two physiological states, that is, corresponding to cell growth and starvation-induced aggregation. In addition, for the first time, Raman spectra have been obtained on human urine exosomes in a preliminary RTM study. Our results suggest RTM as a novel promising tool for a rapid characterisation of EVs of various origins and possibly in the future for fast biochemical diseases diagnosis in physiological fluids.
